# Transcriptomic Analysis of Gene Expression and Effect of Electromagnetic Field in Brain Tissue after Traumatic Brain Injury

**DOI:** 10.26502/jbb.2642-91280131

**Published:** 2024-02-13

**Authors:** Vikrant Rai, Yssel Mendoza-Mari, James Brazdzionis, Mohamed M Radwan, David A Connett, Dan E Miulli, Devendra K Agrawal

**Affiliations:** Department of Translational Research, College of Osteopathic Medicine of the Pacific, Western University of Health Sciences, Pomona CA 91766, USA

**Keywords:** Cell migration, Differentially expressed genes, Electromagnetic field, Inflammation, Oxidative stress, RNA sequencing, Transcriptomic analysis, Traumatic brain injury

## Abstract

Traumatic brain injury (TBI) due to a direct blow or penetrating injury to the head damages the brain tissue and affects brain function. Primary and secondary damage to the brain tissue increases disability, morbidity, and mortality and costs millions of dollars in treatment. Injury to the brain tissue results in the activation of various inflammatory and repair pathways involving many cellular and molecular factors. Increased infiltration of immune cells to clear the debris and lesion healing, activation of Schwann cells, myelination, oligodendrocyte formation, and axonal regeneration occur after TBI to regenerate the tissue. However, secondary damage to brain tissue results in behavioral symptoms. Repair and regeneration are regulated by a complex cascade involving various cells, hormones, and proteins. A change in the expression of various proteins due to altered gene expression may be the cause of impaired repair and the sequelae in TBI. In this pilot study, we used a Yucatan miniswine model of TBI with and without electromagnetic field (EMF) stimulation and investigated the differential gene expression between injured and non-injured cortex tissues. We found several differentially expressed genes including INSC, TTR, CFAP126, SEMA3F, CALB1, CDH19, and SERPINE1. These genes are associated with immune cell infiltration, myelination, reactive oxygen species regulation, thyroid hormone transportation, cell proliferation, and cell migration. There was a time-dependent effect of EMF stimulation on the gene and protein expression. The findings support the beneficial effect of EMF stimulation in the repair process following TBI.

## Introduction

Traumatic brain injury (TBI) either due to a blunt trauma or a penetrating injury affects approximately 1.7 million people in the United States [1]. TBI remains a leading cause of disability, morbidity, and mortality [2]. The underlying pathophysiology remains elusive and warrants in-depth investigation using an animal model. Acute insult to the brain results in parenchymal damage (primary effects) including bleeding, nerve fiber injury, diffuse axonal injury, metabolic alteration, inflammation, and swelling. Secondary injuries occur after primary insult due to the activation of various cellular and molecular processes causing behavioral changes, dizziness, vision problems, slurred speech, memory issues, headaches, etc. [1–3]. Post-injury, activation of lymphocytes, upregulation of adhesion molecules, reactive oxygen species (ROS) production, and secretion of cytokines and chemokines contribute to demyelination and disruption of axons, axonal swelling, and transport proteins accumulation at the axon terminals ultimately causing neurodegeneration [2]. These processes are regulated by a complex cascade of molecular and cellular events regulating cell proliferation, cell survival, inflammation, and neuronal function [4]. Further, these processes are regulated by various proteins encoded by genes. This suggests that changing gene expression will differentially regulate cellular and molecular events. Since dysregulated gene expression is associated with TBI and altered gene expression may have a diagnostic and therapeutic role [5], this pilot study was designed to delineate the differentially expressed genes after TBI and the effect of electromagnetic field (EMF) on gene expression profile. In a recently developed TBI swine model [6–8], we conducted the RNA sequencing using the tissue samples from the injured site and compared the expression profile with the non-injured site (contralateral hemisphere).

## Material and Methods

### Animal model and tissue collection

The protocol (#R23IACUC003) for this study was approved by the Institutional Animal Care and Use Committee of the Western University of Health Sciences, Pomona CA. Male Yucatan miniswine purchased from Premier BioSource (Romona, CA) and kept with 12 hours of light and dark cycle at a temperature range of 72°–74° F at the animal care facility of Western University of Health Sciences, Pomona CA were fed with the Mini-Pig Grower Diet (Test Diet # 5801) and water ad *libitum*. Brain injury was induced, and EMF was applied to one swine after 2 days post-TBI (hereafter swine 2), one swine just after injury (hereafter swine 3), and no EMF in one swine (hereafter swine 1) as described [6–8]. The tissues from the injured and non-injured sites were collected after the swine was euthanized. The tissues were collected in 10% Formalin, in RNA later, and dry freeze for histological analysis, and RNA and protein isolation, respectively. The tissues in RNA later for RNA sequencing were first kept at 4^0^C followed by −20°C and then at −80°C.

### RNA sequencing and data analysis

The RNA sequencing was done at the University of California at Los Angeles (UCLA) using three controls and three injured site tissues. The RNA samples with RIN >6 were used for sequencing and RNA-seq libraries were prepared using KAPA Stranded mRNA-Seq Kit. After mRNA enrichment and fragmentation and first-strand cDNA synthesis using random priming, second-strand synthesis converting cDNA: RNA hybrid to double-stranded cDNA (dscDNA) and incorporating dUTP into the second cDNA strand was done. This was followed by end repair to generate blunt ends, A-tailing, adaptor ligation, and PCR amplification. Different adaptors were used for multiplexing samples in a half-lane. Sequencing was performed on Illumina Novaseq 6000 for the PE 2×50 run. A data quality check was done on Illumina SAV. Demultiplexing was performed with Illumina Bcl2fastq v2.19.1.403 software. The alignment with pig reference genome Sscrofa11.1 was performed using STAR [9]. The Ensembl Transcripts release Sus_scrofa. Sscrofa11.1.110 GTF was used for gene feature annotation. CPM normalized counts were generated by adding 1.0E-4 followed by counts per million (CPM) for normalization of transcripts counts. Partek Flow GSA algorithm (Partek^®^ Flow^®^ software, version 7.0 Copyright ©; 2019 Partek Inc., St. Louis, MO, USA.) was used to determine differential gene expression. A p-value of 0.05 and log_2_ Fold change were used to filter for differentially expressed gene lists. A p-value of 0.05 and 2-fold change was used to filter for differentially expressed genes used for subsequent heatmap generation.

### Real-Time Polymerase Chain Reaction (RT-PCR)

Total RNA extracted using TRIZOL (Sigma #T9424) was subjected to cDNA preparation using Azura 1-Step Ultra RT-PCR Kit (AZ-1825). RT-PCR was conducted in triplicates using AzuraView GreenFast qPCR Blue Mix HR using CFX96 Touch Real-Time PCR Detection System. The nucleotides (Table 1) used for various genes were purchased from Integrated DNA Technology (IDT, Coralville, IA). The cycling conditions were: 5-minute cycling at 95°C for initial denaturation, 39 cycles of 30s at 95°C for denaturation, 30s at 55–60°C for annealing, and 30s cycle at 72°C for extension. This was followed by melting curve analysis. The fold changes in mRNA expression were analyzed using 2^−^ĈT^ method after normalizing with housekeeping gene 18S.

### Immunohistochemistry (IHC)

Tissue sections were deparaffinized and rehydrated using several changes of xylene and graded ethanol. Antigen retrieval was performed using a 1% citrate buffer (Sigma Aldrich #C9999) followed by cooling to room temperature and washing the slides with 1x phosphate-buffered saline (PBS). The tissues were encircled using Pap Pen and incubated with 3% hydrogen peroxide (Sigma Aldrich #H1009) for 15 minutes and then washed with PBS for 5 minutes 3 times each. Vectastain Elite ABC kit (Vector Labs) blocking solution was used for blocking nonspecific antigens for 1 hour at room temperature followed by incubation with primary antibodies (Table 2) overnight at 4^0^C. After overnight incubation, the slides were washed 3 times for 5 minutes each with 1x PBS followed by incubation with the secondary antibody (Table 2) for 1 hour at room temperature. The slides were rinsed 3 times with 1x PBS and incubated with the Vectastain ABC horseradish peroxidase (HRP) for 30 minutes at room temp, followed by 1x PBS wash. The sections were incubated with 3,3′-diaminobenzidine (DAB) (Thermo Scientific, Cat# 34002) for 2–5 minutes until the development of the brown color of the DAB. Tissue sections were washed with water and then counterstained with hematoxylin for 20–30 seconds and mounted with a xylene-based mounting medium. The stained slides were scanned with a Leica DM6 microscope at a scale of 100μm and images from each tissue section were manually analyzed for average stained intensity and percent-stained area using Fiji Image J. A minimum of three sections for each swine were used for statistical analysis.

## Results and Discussion

### RNA sequencing analysis revealed differentially expressed genes (DEGs)

**Figure 1:**
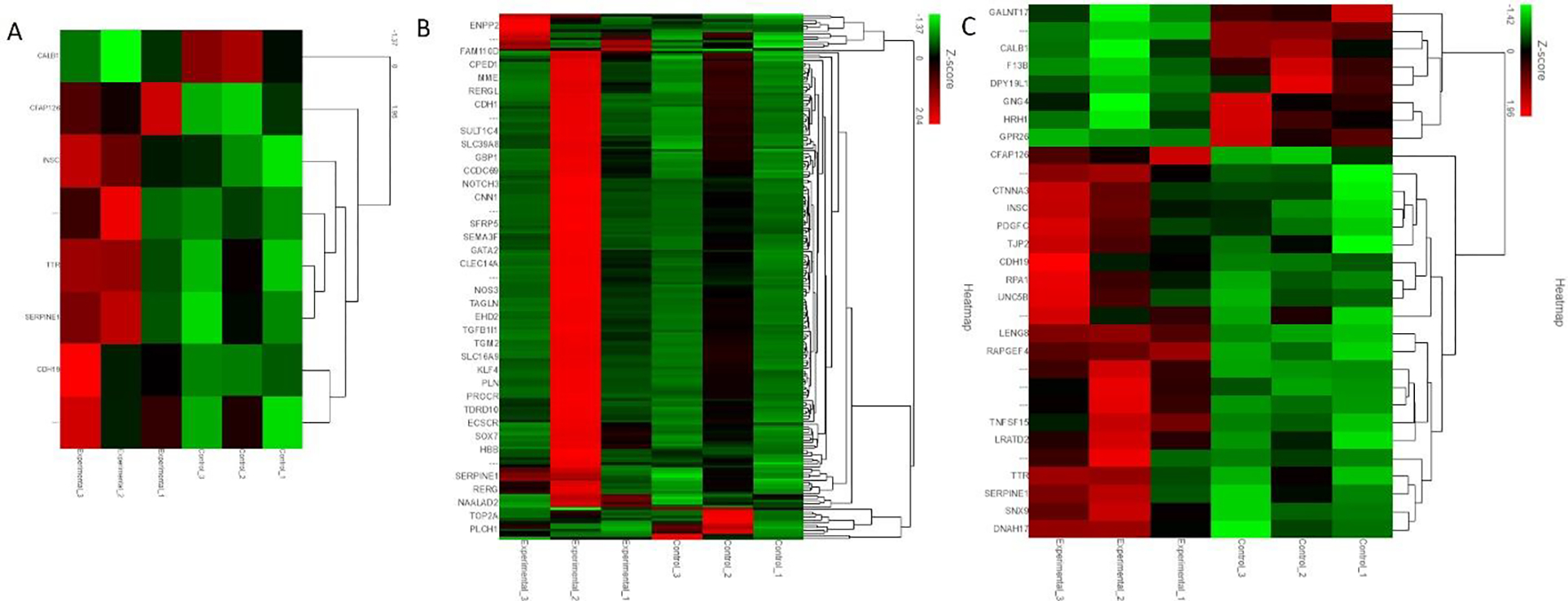
Differentially expressed gene in control tissue (non-injured) compared to injured tissues in the brain cortex of Yucatan miniswine with traumatic brain injury (TBI).

**Figure 2:**
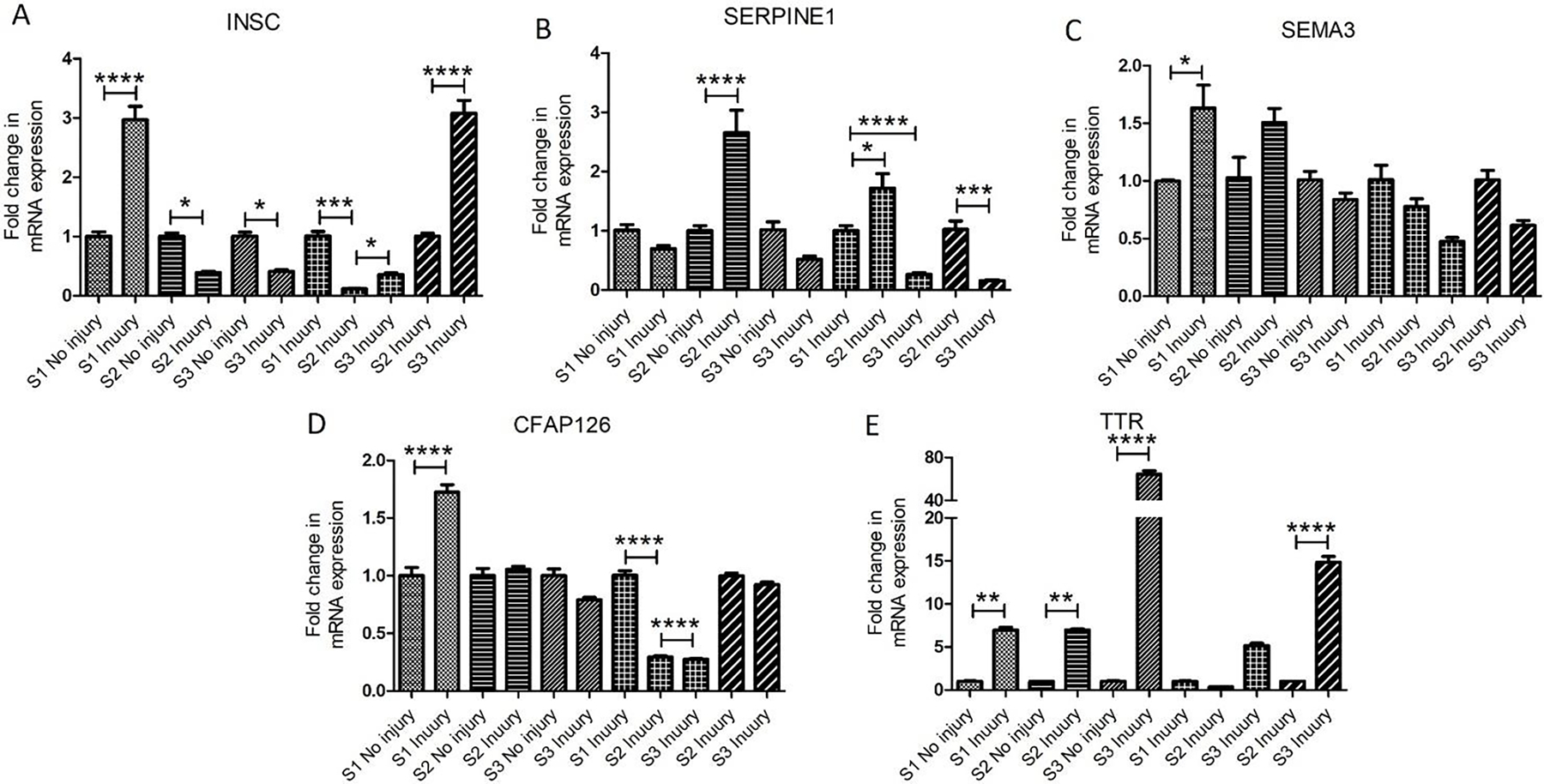
Real-Time Polymerase Chain Reaction for mRNA transcript expression of various genes in non-injured and injured tissues of the brain in Yucatan miniswine.

The analysis of the fold change (average of Cs/average of Es) revealed 8 genes with more than 2-fold change and p<0.05 (Figure 1 panel A). Thirty-nine genes were found to have more than 2-fold change (Figure 1 panel B), and 30 genes with p<0.05 (Figure 2 panel C).

Among the genes with more than 2-fold change and p<0.05, cilia and flagella-associated protein 126 (CFAP126) is involved in cilium organization. The differential expression of CFAP126 has been reported in choroid plexus epithelial cells treated with Pam3CSK4 (P3C), a toll-like receptor (TLR)2 agonist, and lipopolysaccharides (LPS) activating TLR-2 playing a role in cytoskeleton remodeling in leukocyte trafficking [10]. An increased expression of CFAP126 in the injured tissues suggests its involvement in leukocyte trafficking after TBI. Further, an increased CFAP126 in swine 1 with no EMF (experiment 1, Figure 1 panel A) was found compared to swine 2 and 3 with EMF applied after 2 days and immediately after TBI, respectively (experiment 2 and 3, Figure 1 panel A), and to controls without any injury. A decreased expression with EMF suggests the beneficial effect of the treatment because its application will lead to injury repair and decreased leukocyte infiltration (injured lesion healing entering to proliferative or resolution phase after inflammatory phase) because tissues were collected 6–8 weeks after TBI. CFAP126 was also differentially expressed in single-cell profiling of induced pluripotent stem cell-derived cerebellar organoids [11]. Inscuteable (INSC) Spindle Orientation Adaptor Protein is another protein-coding gene that is involved in spindle orientation during mitosis, cell fate decision, regulates cell proliferation and differentiation, in the developing nervous system, and may function as an adapter linking the Par3 complex to the GPSM1/GPSM2 complex [12, 13]. INSC expression significantly correlates negatively with the infiltration levels of various immune cells including B cells, CD4+ T cells, macrophages, and dendritic cells (DCs) [14]. An increased expression of INSC after TBI in our study (Figure 1 panel A) compared to controls suggests cell proliferation and differentiation after TBI. Further, a differential expression of INSC between swine 1, 2, and 3 indicates the effect of EMF. An increased expression in swine 3 compared to swine 2 and swine 1 and in swine 2 compared to swine 1 in injured tissues suggest an increased immune cell infiltration with EMF application and the possibility of the brain lesion entering to proliferative or resolving phase.

Transthyretin (TTR) was increased in swine 2 and swine 3 compared to swine 1 in experimental tissues as well as compared to uninjured tissues in all three swine. TTR is involved in the transport of thyroid hormone thyroxine and retinol-binding protein (RBP) bound to retinol (vitamin A). TTR is associated with enhanced recruitment of muscle satellite cells at the injury site, promotes myoblast proliferation, and regulates muscle regeneration [15]. TTR is involved in the development of oligodendrocytes [16] and posttraumatic regeneration of the sciatic nerve [17]. Further, thyroid hormone is essential for myelination (proliferation of myelin-producing cells) and oligodendrocyte maturation [18]. An increased expression in injured tissues of swine 2 and swine 3 after EMF application suggests the beneficial role of EMF in promoting oligodendrocyte proliferation and myelination, both required for the repair of the injured tissue. SERPINE 1 (serine proteinase inhibitor (serpin) family E member 1) encoding for protein plasminogen activator inhibitor 1 (PAI-1) is involved in hemostasis (normal blood clotting) after an injury as a powerful procoagulant by inhibiting tPA/uPA, thrombomodulin, and activated protein C [19, 20]. PAI-1 also regulates immune cell infiltration [21]. PAI-1 is activated by the production of reactive oxygen species (ROS) [22] whose production increases after TBI [23]. PAI-1 increases tissue damage after TBI by impairing fibrinolysis and clot formation in the microvasculature [24]. A decreased SERPINE1 expression in swine 3 compared to swine 2 indicates the protective effect of EMF when it is applied immediately after an injury compared to its application 2-days after injury in our swine model. However, an increased expression in swine 1 compared to controls but less than swine 2 and swine 3 raises concerns because swine 1 did not receive EMF, and ROS levels should be high. Further, increased PAI-1 is associated with clot formation in microvasculature, and this may be detrimental as a secondary damage after TBI. This interpretation is based on only one swine in each group and thus, conducting these experiments with an increased number of swine is warranted.

CDH19 (cadherin 19) encodes calcium-dependent cell-cell adhesion glycoprotein containing five extracellular cadherin repeats (Figure 1 panel A). CDH19 is a neural crest stem cell marker and is involved in myelin formation [25, 26]. An increased expression of CDH19 in experimental animals (injured tissues) compared to control animals (control tissues) indicates that CDH19 expression increases after TBI to promote myelination and axonal repair. Further, an increased CDH19 expression in swine 3 (EMF just after injury) compared to swine 2 (EMF 2 days after injury) and swine 1 (no EMF) suggests the beneficial effect of EMF in promoting myelination. While analyzing the RNA seq data, we found that another cadherin protein CDH11 (cadherin 11), though not upregulated while comparing the overall raw gene counts, however, was increased after EMF in swine 3 compared to swine 1 and swine 2 (fold change was 1.09, 0.71, and 1.5 in swine 1, 2, and 3 respectively). This suggests that CDH11, which encodes type II classical cadherin, an integral membrane protein mediating calcium-dependent cell-cell adhesion may be an attractive target because CDH11 regulates immune response. CDH11 and CDH9 expression has been shown in the mouse cerebellum using *in-situ* hybridization [27]. CDH11 is significantly expressed after TBI [28]. Loss of CDH11 is associated with altered immune response, impaired (decreased) macrophage migration, and altered T-cell activation [29]. This suggests that decreased expression of CDH11 will result in altered immune response and may result in enhanced injury while increasing its expression will be beneficial. CALB1 (Calbindin 1) encodes a protein that is a member of the calcium-binding protein superfamily that includes calmodulin and troponin C. A decreased expression of CALB1 in the hippocampus after hypoxia-ischemic injury due to astrogliosis is associated with memory impairment [30]. Chitinase Domain Containing 1 (CHID1), a gene whose expression is strongly associated with CALB1, is differentially expressed in various brain regions including the prefrontal, frontal, occipital, cerebellum, temporal, and limbic systems [31]. This suggests that CALB1 expression may also differ in various regions of the brain. Further, the calcium buffering capabilities of calbindin 1 have been suggested to have neuroprotective effect [32]. A higher expression of CALB1 in control tissues compared to injured (experimental) tissues supports the neuroprotective role of CALB1 while a decreased expression in experimental tissues suggests a need to target CALB1 to avoid further brain injury by increasing CALB1 expression. This notion is supported by the fact that CALB1 expression is increased after EMF was applied just after injury compared to the swine where EMF was applied after 2 days (Figure 1 panel A). These findings also indicate a beneficial effect of EMF.

SEMA3F (semaphorin 3F) is a secreted signaling protein and is involved in axon guidance during neuronal development. SEMA3F, which binds to the receptor neuropilin-2, plays a significant role in the development of limbic system and peripheral nervous system circuitry and neurons need it for selective axonal guidance [33]. Neuropilin-2 binds to vascular endothelial growth factor and regulates angiogenesis and lymphatic vessel growth [34]. SEMA3F is involved in oligodendrocyte precursor recruitment and remyelination in the adult central nervous system and its overexpression promotes oligodendrocyte maturation and remyelination [35]. Further, SEMA3F is also involved in collapsin response mediator protein 2 mediated axon pruning and dendrite remodeling [36]. Collapsin is a protein in the brain involved in the collapse and paralysis of neuronal growth cones [37]. This suggests that SEMA3F may play a critical role in the remyelination and growth of neurons after TBI. The fold change in SEMA3F was 1.54, 3.27, and 2.35 in swine 1, 2, and 3. This suggests that EMF application after 2 days was more effective in increasing SEMA3F expression after TBI compared to EMF application just after injury (Figure 1 panel B).

The analysis for the mRNA transcript expression by RT-PCR revealed differential expression of INSC in all 3 swine. The fold change in mRNA expression was significantly increased at the injury site in swine 1 but significantly decreased in swine 2 and swine 3 compared to the non-injured site (Figure 2 panel A). While comparing only the injured site, the expression was significantly decreased in swine 2 and swine 3 compared to swine 1 while the mRNA expression in swine 3 was significantly increased compared to swine 2. These results suggest that EMF application significantly attenuates INSC expression, but INSC levels are time-dependent as INSC expression significantly increases if EMF is applied just after injury compared to EMF applied 2-days after injury. An increase in INSC after injury in swine 1 indicates an increased infiltration of immune cells and a decrease in INSC expression after EMF may be due to decreased cell proliferation at the time of tissue collection as the lesion healing is enhanced by EMF application. However, the cause for a significant difference between swine 2 and swine 3 warrants more in-depth research. The expression pattern of INSC in RT-PCR followed the same pattern as observed in RNA sequencing.

The fold change in mRNA expression of SEMA3 was increased in injury site tissues compared to non-injured site tissues in swine 1 and swine 2 compared to swine 3 which showed decreased a decrease in mRNA expression. This may be because of EMF application just after injury having a healing effect of EMF which is not the case in swine 1 and swine 2 and tissue repair is delayed. The SEMA3 mRNA expression levels follow the same trend when only injured site tissues were compared (Figure 2 panel B). The mRNA expression of SERPINE1 in swine 2 was significantly higher in the injured tissue compared to non-injured tissue and injured tissues of swine 1 and swine 3 (Figure 2 panel C). While comparing the injury site only, the mRNA expression in swine 1 was higher than in swine 3. This indicates an attenuating effect of EMF in reducing the ROS levels in swine 3, however, significantly higher levels of ROS in swine 2 in which EMF was applied 2 days after injury raises concerns and needs more swine to reach any conclusion. Additional studies with increased sample size are required because of the inconsistencies between the RNAseq and RT-PCR results. The mRNA expression of CFAP126 was significantly higher in swine 1 while nearly the same in swine 2 and decreased in swine 3 in the injured tissues compared to non-injured tissues. The nearly same and decreased mRNA expression of CFAP126 in swine 2 and 3 suggest the effect of EMF in decreasing CFAP126 expression (Figure 2 panel D). These findings corroborate the findings of RNA seq results as discussed above. TTR’s mRNA expression was significantly higher in swine 1, 2, and 3 in the injured tissues compared to non-injured tissues (Figure 2 panel E). On comparing the injured tissues, the mRNA expression was significantly higher in swine 3 compared to swine 2 and swine 1. TTR is involved in myoblast proliferation and regulates muscle regeneration [15], the development of oligodendrocytes [16], posttraumatic regeneration of the sciatic nerve [17], and myelination (proliferation of myelin-producing cells) and oligodendrocyte maturation [18] by promoting thyroid hormone. An increased TTR expression after injury and more with EMF suggests that EMF application enhances neuronal repair and myelination. These findings corroborate the findings of RNA sequencing.

**Figure 3:**
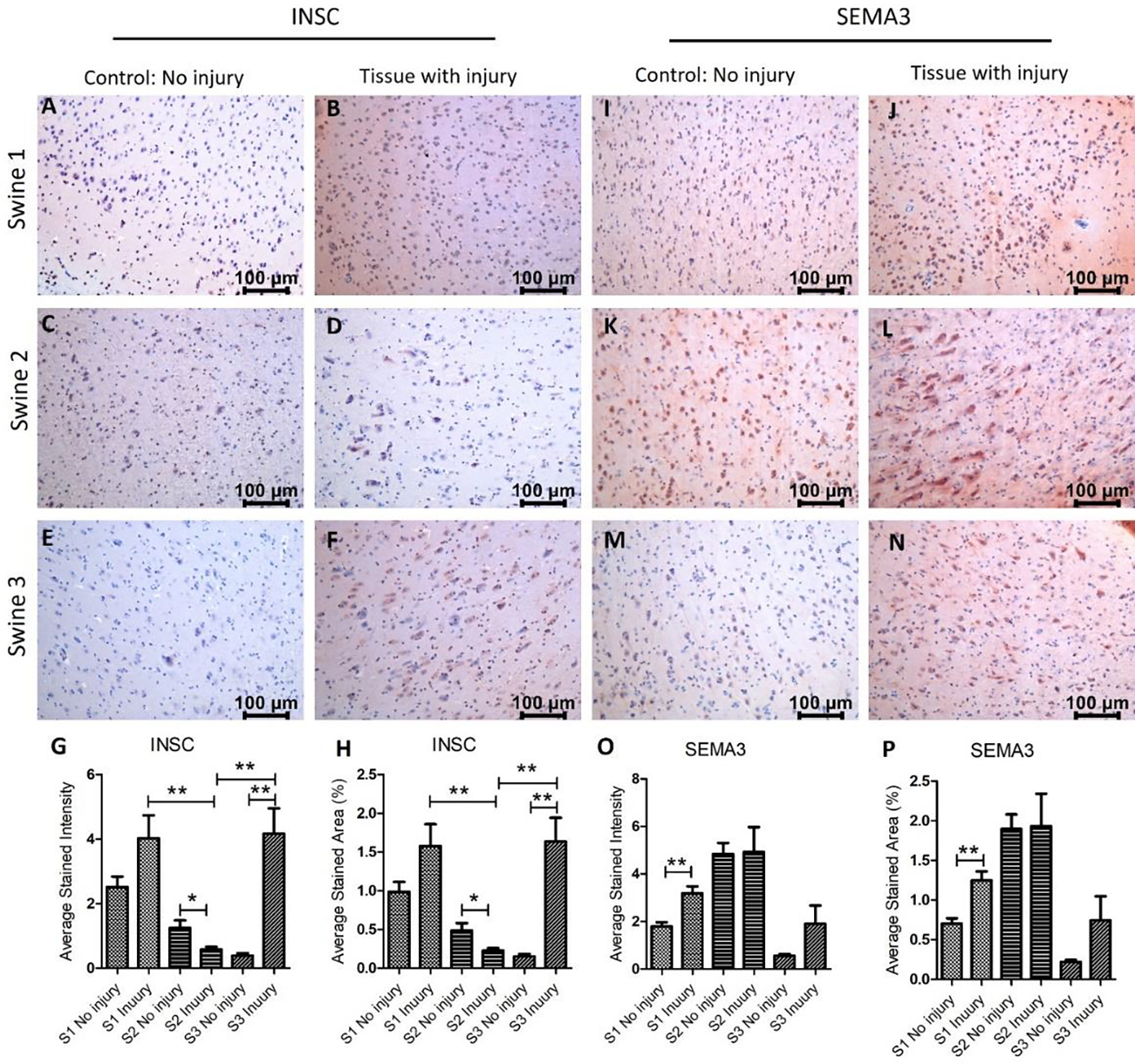
Immunohistochemistry (IHC) staining for inscuteable (INSC) and semaphorin (SEMA)3 in non-injured and injured cortex tissues of Yucatan miniswine. These images are representative of all three swine involved in this study. Data is presented as mean ± SD and p<0.05 is considered as significant. *p<0.05 and **p<0.01. IHC for INSC (panels A-F), SEMA3 (panels I-N), average stained intensity (panels G and O), and average stained percent area (panels H and P).

Next, we investigated the protein expression of INSC, SEMA3, SERPINE1 (PAI-1), and TTR in the non-injured and injured tissues. IHC results revealed that INSC expression was significantly increased in swine 1 and swine 3 in the injured tissues compared to non-injured tissues while was significantly lower in injured tissues in swine 2 (Figure 3 panels A-H). The immunopositivity for SEMA3 was significantly higher in swine 1, nearly the same in swine 2, and increased in swine 3 in the injured tissues compared to non-injured tissues (Figure 3 panels I-P). SEMA3F is involved in axon pruning and dendrite remodeling [36] and significantly increased expression in swine 1 suggests enhanced repair after injury in swine 1, however, no effect in swine 2 and a minimal effect in swine 3 after EMF needs further investigation. Although, there was an effect of EMF in swine 3 when applied immediately after injury. This suggests that the beneficial effect of EMF application is time-dependent, and an early application is better.

**Figure 4:**
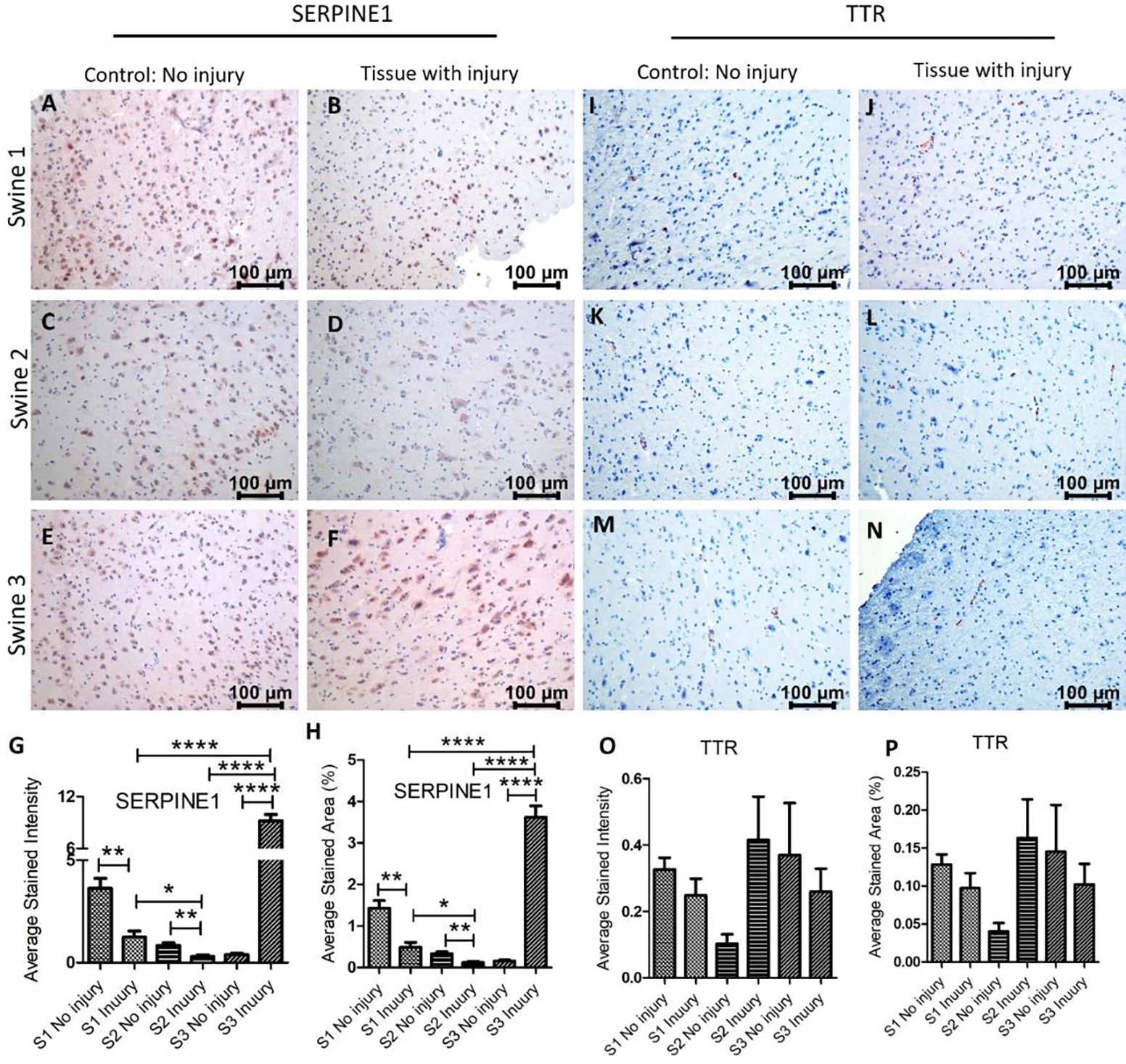
Immunohistochemistry (IHC) staining for serine proteinase inhibitor (serpin) family E member 1 (SERPINE1) and transthyretin (TTR) in non-injured and injured cortex tissues of Yucatan miniswine. These images are representative of all three swine involved in this study. Data is presented as mean ± SD and p<0.05 is considered as significant. *p<0.05 and **p<0.01. IHC for SERPINE1 (panels A-F), TTR (panels I-N), average stained intensity (panels G and O), and average stained percent area (panels H and P).

Immunostaining for SERPINE1 (PAI-1) revealed a significant decrease expression in swine 1 and 2 in the injured tissues compared to non-injured tissues with a significant increase in swine 3. While comparing the injured tissues, mRNA expression in swine 2 was significantly lower than in swine 1 while the expression in swine 3 was significantly higher in injured tissues compared to swine 1 and 2 (Figure 4 panels A-H). Since SERPINE1 is associated with ROS levels and acts as an antithrombotic, an increase after immediate EMF while a decrease in swine with EMF after 2 days warrants further investigation with more swine. The TTR immunopositivity was very minimal in both non-injured and injured tissues and only a few cells show immunopositivity. The immunopositivity was decreased in the injured tissues in swine 1 and 3 while was increased in swine 2 (Figure 4 panels I-P). Comparing the results of RT-PCR and IHC revealed that both, gene and protein expression, of INSC, SERPINE1, and SEMA3F increase after injury in swine 1, and of INSC and TTR decrease after injury in swine 2. However, the protein and gene expression in swine 3 were different. This may be due to only one swine in each group and more swine should be included with similar treatment strategies. Another reason may be the involvement of epigenetic regulation of various genes encoding proteins [38] involved in brain tissue repair and regeneration.

Another potential cause of differentially expressed genes between swine 1 and swine 2 and 3 may be the application of EMF [39]. Since EMF was applied to swine 2 and 3 but not to swine 1, DEGs may be due to EMF but EMF was applied to both swine 2 and swine 3 and on different time points. Differential gene expression in these two swine may be due to the difference in the time of application of EMF [40]. The frequency for pre-synaptic and post-synaptic action potentials and cell maintenance ranges between 30–5000 Hz [41]. EMF regulates metabolism and transport of neurotransmitters which play an important role in cognitive and emotional behavior [42]. Additionally, the frequency of EMF is also important, and a low frequency is associated with changed gene expression, stress response, and hormesis [43–45]. A later onset of electrical stimulation is associated with better clinical outcomes. However, electrical stimulation for 7 days after 7 weeks of TBI was found to have no persistent effect [46]. Thus, it is important to investigate the time, frequency, and doses of EMF application for a favorable outcome.

## Conclusion

The results of this pilot study suggest a differential expression of various genes in injured and non-injured tissues after TBI. There was an effect of EMF application on the gene and protein expression and in a few cases, it was time dependent. Since these genes and encoded proteins are involved in regulating immune cell infiltration, myelination, and axonal repair, future studies are warranted with more swine in each group with similar treatment strategies as well as with different frequencies to identify the best frequency, time, and doses to change the gene expression favorable for injured lesion healing, tissue repair, and axonal repair.

## Figures and Tables

**Table 1:** Forward and reverse nucleotide sequence used in RT-PCR.

Gene name	Forward sequence	Reverse sequence
INSC	5’-GCAGTGACAAGCAGAGAGTAG-3’	5’-CCCGTTCTCCTGAATGATGT-3’
TTR	5’-CCATGAATATGCAGAGGTTGTG-3’	5’-GCTGTGGTGGAGTAAGAGTAG-3’
SERPINE1	5’-GCCGTGGAACAAAGATGAGA-3’	5’-CGGAACAGCCTGAAGAAGTAG-3’
SEMA3	5’-GCTCTTCATGCTCTCGCTATT-3’	5’-GAGTCAGTGGGTCTCCATTTC-3’
CFAP126	5’-CAAGCTCCAAGATCAACCATAATC-3’	5’-GGCTTCCAAGTGAGGTCTTT-3’
18S	5’-CCCACGGAATCGAGAAAGAG-3’	5’-TTGACGGAAGGGCACCA-3’

INSC (INSC Spindle Orientation Adaptor Protein), TTR (transthyretin), SERPINE1 (serine proteinase inhibitor (serpin) family E member 1), SEMA3 (Semaphorin 3), and CFAP126 (Cilia and Flagella Associated Protein 126).

**Table 2:** Primary and secondary antibodies used in this study.

Antibody	Catalog	Dilution in IHC	Dilution in WB
**Primary antibodies**			
INSC	ABIN653656	1:50	1:1000
TTR	MBS2027685	1:50	1:2000
SERPINE1	66261-I-Ig	1:250	1:5000
SEMA3	Orb475025	1:100	1:1000
ACTB	ab8226	-	1:1000
**Secondary antibodies**			
Anti-mouse	BP-2000-50	Ready-to-use	-
Anti-rabbit	BP-9100-50	Ready-to-use	-
Anti-mouse	NB7544	-	1:3000
Anti-rabbit	A16023	-	1:2000

INSC (INSC Spindle Orientation Adaptor Protein), TTR (transthyretin), SERPINE1 (serine proteinase inhibitor (serpin) family E member 1), SEMA3 (Semaphorin 3), and ACTB (beta-actin).
